# Weakening or Structural Strengthening? An Evaluation of Bone Density after MRgFUS Ablation for Treatment of Benign Bone Lesions

**DOI:** 10.3390/jcm11010182

**Published:** 2021-12-29

**Authors:** Camilla de Cataldo, Federico Bruno, Stefano Necozione, Mariangela Novello, Pierpaolo Palumbo, Luigi Zugaro, Antonio Barile, Carlo Masciocchi, Francesco Arrigoni

**Affiliations:** 1 Department of Emergency and Interventional Radiology, San Salvatore Hospital, 67100 L’Aquila, Italy; federico.bruno1988@gmail.com (F.B.); palumbopierpaolo89@gmail.com (P.P.); luigi.zugarodoc@gmail.com (L.Z.); arrigoni.francesco@gmail.com (F.A.); 2 Epidemiology Unit, Department of Life, Health and Environmental Science, University of L’Aquila, 67100 L’Aquila, Italy; stefano.necozione@univaq.it; 3 Department of Pathology, IRCSS Regina Elena National Cancer Institute, 00144 Rome, Italy; mariangelanv@gmail.com; 4 Department of Biotechnological and Applied Clinical Sciences, University of L’Aquila, 67100 L’Aquila, Italy; abarile63@gmail.com (A.B.); masciocchi.carlo@gmail.com (C.M.)

**Keywords:** osteoid osteoma, osteoblastoma, MRgFUS, bone density, ablation, musculoskeletal interventional radiology

## Abstract

Previous studies suggest that interventional ablative procedures on bone lesions may weaken the bone, especially when performed through the needle approach. Our purpose was to evaluate, through Computed Tomography (CT), the effects of Magnetic Resonance guided Focused Ultrasound Surgery (MRgFUS) ablation on painful osteoid osteomas and osteoblastomas in terms of bone density and morphological changes. We retrospectively evaluated patients treated at our institution with MRgFUS for superficial, painful osteoid osteoma or osteoblastoma during the last 9 years. Inclusion criteria were procedural and clinical success, as well as the availability of pre- and postprocedural CT examinations. Imaging features assessed were perilesional/nidus density changes and the occurrence of pathological fractures during the follow-up period. Our study population included 31 osteoid osteomas and 5 intra-articular osteoblastomas in 36 treated patients. We found an increased bone density of the lesions when pre and post-treatment CT- values were compared: these differences were statistically significant, and this finding is consistent with significant bone densification at the post-treatment imaging follow-up. No pathological fractures were observed after ablation during the follow-up. MRgFUS can be considered to be the treatment of choice for benign superficial bone lesions, thanks to its minimal invasiveness, excellent effectiveness, and safety. Pathological fractures, reported in literature as a rare event using needle ablation, never occurred in our MRgFUS treatment series.

## 1. Introduction

Osteoid osteoma (OO) and Osteoblastoma (OB) are benign bone-forming tumors, representing the most common focal and painful benign bone lesions. Their clinical relevance consists in their disabling symptomatology, which is typically responsive to non-steroidal anti-inflammatory drugs (NSAIDS), and functional impairment with a significant social effect, considering their high incidence among young people, without neglecting the possibility of malignant transformation described in the literature, representing 12–25% of OB [1].

The therapeutic management of OO and OB is challenging, as the rare possibility of self-healing OO has also been described [2]. What is out of the question nowadays is that the main operative approach to these lesions falls into the field of Interventional Radiology, due to its higher success rates and lower invasiveness when compared to surgery. Conservative treatments do not represent a reasonable solution, due to the long-term drug therapy that is required.

Among the ablative techniques used in Interventional Radiology, RFA is considered the gold standard in the treatment of these painful lesions, due to its technical spread and availability, uncontested reliability, and efficacy. Though it is minimally invasive, RFA brings some complications, including post-procedural bone fractures, which are rare, but disabling [3,4,5]. The main cause of post-operative fractures is controversial, whether they are related to bone weakening caused by needle access or to the effect of the thermal ablation on bone tissues. In this scenario, Magnetic Resonance guided Focused Ultrasound Surgery (MRgFUS) is also involved as a promising ablative technique, representing an interesting alternative to RFA. It is a needle-free, thus minimally invasive, ablative technique and its effectiveness and safety are comparable to RFA. One of its shortcomings, however, is that it is suitable only for superficial lesions, due to its physical mechanisms [6,7,8].

To contribute to this debate, in this study we evaluated the bone density changes (through Computed Tomography, CT) within the area of treatment of painful osteoid osteomas and osteoblastomas (managed using Magnetic Resonance guided Focused Ultrasound Surgery, MRgFUS) during follow-up, in order to describe the effect of thermal ablation on the bone in vivo.

## 2. Materials and Methods

### 2.1. Patients

We retrospectively evaluated patients treated with MRgFUS for superficial painful osteoid osteoma or osteoblastoma at our musculoskeletal interventional center between January 2012 and February 2021. Inclusion criteria were the procedural and clinical success and availability of pre- and post-procedural CT examinations performed at our musculoskeletal center. CT examinations were carried out using a 640-slice CT (Toshiba Aquilion ONE 320-row detector of 0.5 mm each), before and after treatment, during the regularly scheduled follow-up. Exclusion criteria were: (1) unavailability of CT images during follow-up at our Institution; (2) disease recurrence or MRgFUS procedural failure, due to an inadequate acoustic window.

### 2.2. Imaging Analysis

**Figure 1:**
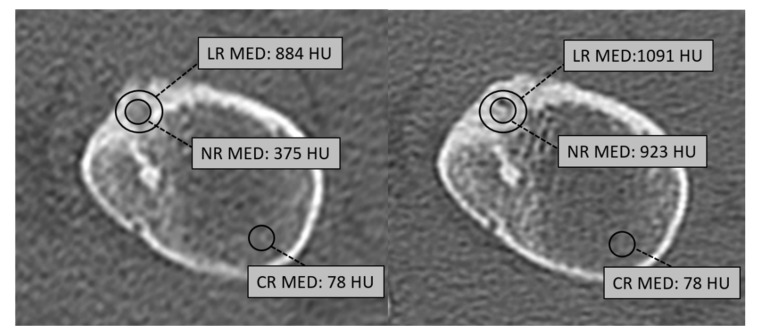
We manually traced, in the most reproducible way, three different ROIs for nidus (Nidus ROI, NR), whole lesion with perilesional sclerosis (Lesion ROI, LR) and a control area (Control ROI, CR) on a nearby normal trabecular bone area.

We analyzed CT images of all the patients submitted to MRgFUS for the treatment of osteoid osteomas/osteoblastomas using the PACS workstation (Carestream v.12, Philips). On both pre-treatment and post-treatment CT examinations, we manually traced, in the most possibly reproducible way, three ovoidal or circular regions of interest (ROIs) on axial planes including (1) nidus of OO/OB (nidus ROI, NR); (2) whole lesion area, including perilesional sclerosis, with a global diameter equal to twice the nidus diameter (Lesion ROI, LR); (3) a normal trabecular bone area not included in the volume of treatment with the same nidus diameter, traced on the same slice of the other ROIs (Control area ROI, CR) (Figure 1). For each ROI, we recorded size and density values; the occurrence of pathological fractures during the follow-up period was also recorded.

### 2.3. Statistical Analysis

Data analysis was performed using the SAS software (SAS Institute, Cary, NC, USA, version 9.4). A descriptive analysis, consisting of distribution statistics (number of available observations, mean, standard deviations, median, minimum, and maximum), were presented for continuous data.

The Shapiro-Wilk test was used to test the normality of the variables’ distribution.

A two-factor ANOVA with repeated measures on one factor, after logarithmic transformation in consideration of the non-normal distribution of the data, was used to evaluate differences of time in the values of variables in the treated bone area and the normal bone control area. Pre- and post-treatment differences in the treated bone area and normal bone control area were also evaluated with the Wilcoxon Test for paired data.

## 3. Results

Our final study population consisted of 36 patients affected by osteoid osteomas in 31 cases and intra-articular osteoblastomas in five cases (22 males, 14 females, mean age at the time of treatment 23.67 ± 10.44 years). The post-procedural follow-up was 18 ± 6 months. 

**Figure 2:**
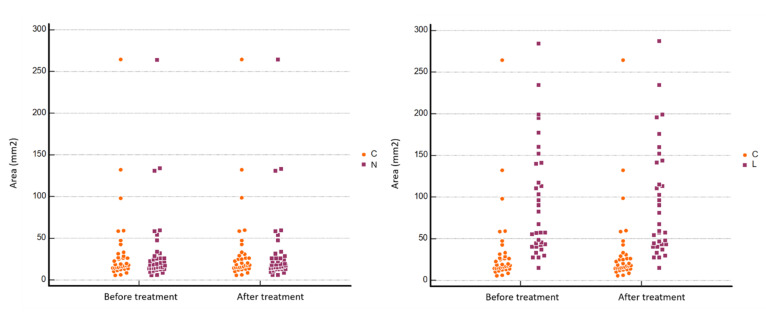
Accuracy of manually traced nidus ROIs (NR) and Lesion ROIs (LR) when compared to Control ROIs (CR) both on pre and post-treatment evaluation. There were no statistically significant differences in ROIs’ area measured (two-factor ANOVA with repeated measures on one factor, CI = 95%) on pre and post-treatment CT examinations. Y axis: ROIs’ area (C: Control ROI area; L: Lesion ROI area; N: Nidus ROI area).

**Figure 3:**
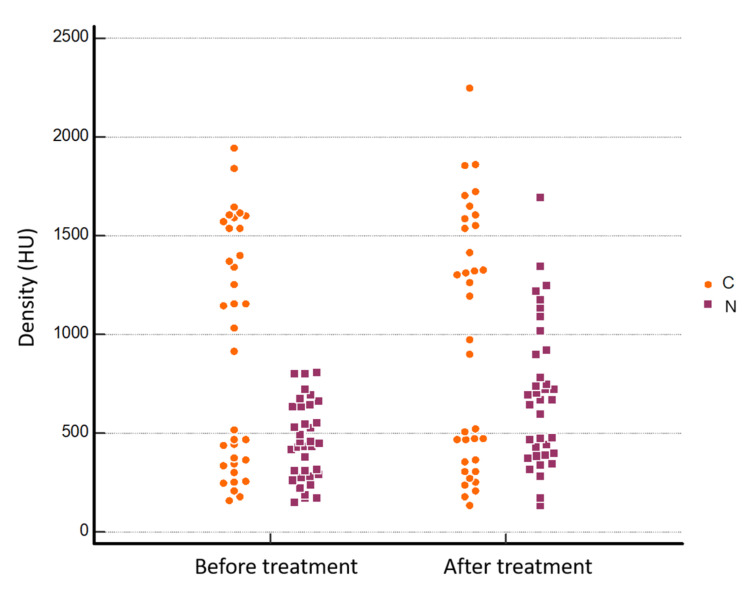
Bone density values at nidus level (NR) before and after MRgFUS treatment (452.75 ± 195.31 HU and 688.14 ± 362.45 HU, respectively, treatment*time interaction *p* < 0.001, estimated with two-factor ANOVA with repeated measures on one factor, CI = 95%). Y axis: bone density; N: nidus ROI; C: control ROI.

**Figure 4:**
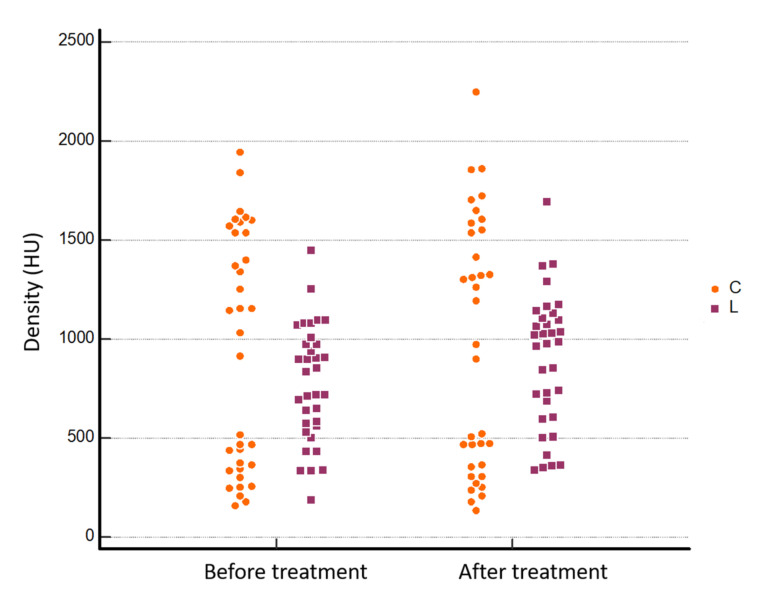
Bone density values at lesion level (LR) before and after MRgFUS treatment (770.50 ± 293.99 HU and 890.91 ± 338.09 HU, respectively, treatment*time interaction *p* = 0.010, estimated with two-factor ANOVA with repeated measures on one factor, CI = 95%). Y axis: bone density; L: Lesion ROI; C: control ROI.

All the detailed results of the analysis are summarized in Table 1. The areas of the NRs and LRs measured before and after treatment were completely comparable without any statistically significant difference (Figure 2). Regarding CT density values, we found a significant bone densification at the imaging follow-up when the median pre- and post-treatment values were compared: 452.75 ± 195.31 HU vs. 688.14 ± 362.45 HU and 770.50 ± 293.99 HU vs. 890.91 ± 338.09 HU, respectively, for NR and LR (treatment*time interaction *p* < 0.001 and *p* = 0.010, respectively) (Figure 3 and Figure 4). No statistically significant difference was found between pre- and post- treatment density values in the normal bone control area (CR), as the bone density of untreated areas did not show significant modifications during follow-up. No pathological fractures were observed after ablation during the follow-up.

## 4. Discussion

Magnetic Resonance guided Focused Ultrasound Surgery (MRgFUS) ablation is an excellent therapeutic strategy for bone lesions demonstrating its additional strength when compared to needle procedures in its independence from the percutaneous approach. This is particularly helpful in avoiding infective complications, which is of capital importance, especially in case of intra-articular lesions. The main drawback of this technique is that only the lesions on the bone surface can be treated.

**Figure 5:**
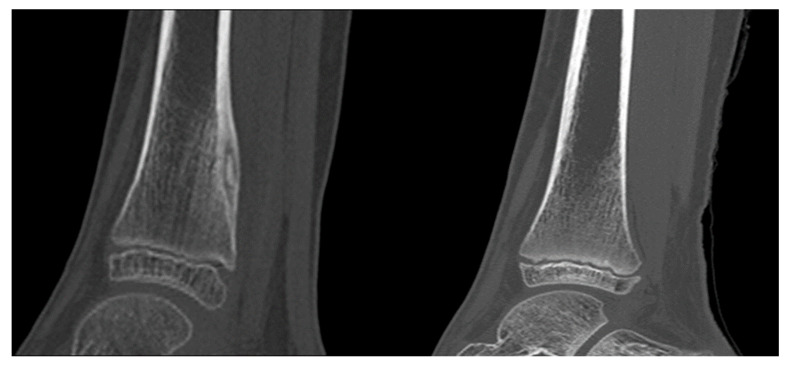
An OO of the distal diaphysis in an 8-years old girl treated successfully with MRgFUS. A complete restitution ad integrum was observed (follow-up time: 24 months).

**Figure 6:**
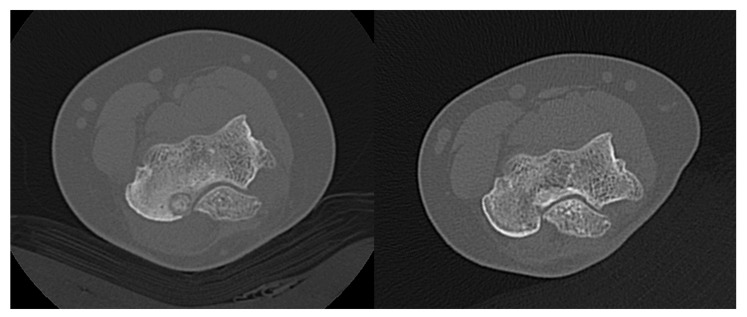
An OB of the elbow in a 16 year-old girl treated successfully with MRgFUS. A complete restitution ad integrum with normal trabecular bone restoration was observed (follow-up time: 22 months).

**Figure 7:**
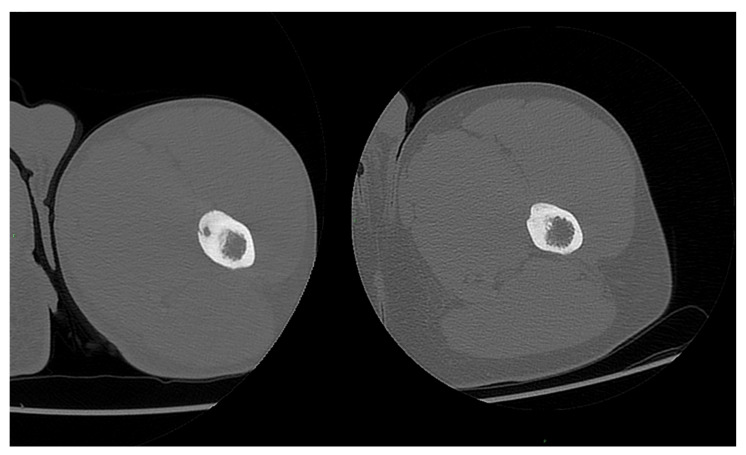
An OO of the proximal diaphysis of the femur in a 19 year-old boy treated successfully with MRgFUS. A complete restitution ad integrum was observed (follow-up time: 18 months).

Pathological fractures are a quite common periprocedural complication after ablative procedures on bone metastases, regardless of the ablation technique employed, due to the usually massive weakening of the bone caused by the pathological tissue; nevertheless, there are few studies reporting bone complications in interventional treatments on benign bone lesions. Measuring a few cubic millimeters or centimeters, the size of the treated volume of these lesions can hardly justify a pathological fracture. Some reports describe fractures as a complication occurring after RFA of benign lesions [9] and, also in the authors’ experience, one case of post-RFA fracture, conservatively managed and healed, in a 20-year-old man with an osteoid osteoma located in the transverse process of a thoracic vertebra, was observed. This potential periprocedural risk, together with damage of soft tissues, critical structures, and skin, due to hyperthermia, represents a real possible complication in the therapeutic strategy. In a previous paper, nevertheless [1], the authors describe a progressive sclerotic process of the nidus of osteoblastomas treated with RFA. In another study [10], based on a larger series of lesions (Osteoid Osteomas) treated with RFA and MRgFUS, the authors also demonstrate bone remodeling with ossification. In particular, the authors set 12 months after treatment as the optimal time for follow-up to see the initial signs of ossification. To our knowledge, however, in the literature, there is no assessment of bone modifications in vivo after ablation of OB and OO, which, conversely, have been observed in swine models [11,12]. Also, quantitative evaluations of the bone segment treated have never been made. The purpose of this study was an objective quantification of post-treatment bone changes accompanying morphological CT modifications. To evaluate the strength of the bone, we decided to calculate the bone density of the focal lesion (the nidus) before and after treatment. Measurements were performed only in patients ablated with MRgFUS, to avoid confounding factors that would result by the presence of the needle tract in the bone. A statistically significant bone densification was observed following MRgFUS ablation, both in OO and OB, at the nidus as well as at the perilesional level. We evaluated the normal bone density values of physiological bone near the lesion and noticed that these measurements also varied in the follow-up studies, without reaching statistical significance. This phenomenon was probably related to the normal growth and mineralization of bone in young patients. When the values describing density modification before and long after the procedure were compared, they showed a statistically significant difference, which supports the thesis that a successful treatment can destroy the pathological tissue and induce a restitutio ad integrum of the affected bone segment (Figure 5, Figure 6 and Figure 7). This phenomenon is particularly evident in these kind of lesions, due to their usually osteolytic nature. The lack of significant statistical differences between the areas of NR and LR before and after treatment means that, although the segmentation was manually performed, it was accurate and the results were reliable.

Our study was limited by the small specimen size. Although the study was developed in our musculoskeletal interventional center, which collects patients from different Italian regions, we decided to include only those patients with instrumental examinations from diagnosis to follow-up, which were performed in our institution, to avoid possible measurement bias due to different CT scanner characteristics. Moreover, this was only a quantitative study based on CT imaging: no structural or biomechanical evaluations were performed.

## 5. Conclusions

MRgFUS can be considered the treatment of choice for benign superficial bone lesions, thanks to its minimal invasiveness, excellent effectiveness, and safety. Our report has demonstrated a physiological increase of bone density of the osteolytic lesion after treatment that leads to a strengthening of the bone segment. The occurrence of pathological fractures, rarely reported in literature with needle ablation, was never encountered in our MRgFUS treatment series.

## Figures and Tables

**Table 1:** Population and Imaging characteristics.

Variable	N	Mean	Std Dev	Minimum	Median	Maximum
Age	36	23.67	10.44	8.00	21.00	60.00
Nidus diameter (mm)	36	6.06	2.94	2.00	6.00	15.00
Nidus ROI before treatment (mm^2^)	36	34.80	48.70	5.40	18.60	263.30
Nidus density before treatment (HU)	36	452.75	195.31	145.00	438.50	806.00
Lesion ROI before treatment (mm^2^)	36	92.81	66.95	14.60	62.10	284.00
Lesion density before treatment (HU)	36	770.50	293.99	185.00	774.50	1445.00
Nidus ROI post treatment (mm^2^)	36	34.82	48.78	5.60	18.55	264.40
Nidus density post treatment (HU)	36	688.14	362.45	133.00	668.50	1689.00
Lesion ROI post treatment (mm^2^)	36	92.90	67.18	14.60	62.80	287.00
Lesion density post treatment (HU)	36	890.91	338.09	338.00	979.50	1691.00
Control Area ROI before treatment (mm^2^)	36	34.12	47.58	5.50	18.20	264.30
Control Area density before treatment (HU)	36	929.34	594.30	157.00	1029.00	1942.00
Control Area ROI after treatment (mm^2^)	36	34.18	47.63	5.50	18.20	264.30
Control Area density after treatment (HU)	36	964.97	630.21	131.00	970.00	2243.00
